# Charting the trajectory of domestic violence policy change in the Republic of Ireland since the mid-1990s – a path towards integration?

**DOI:** 10.5334/ijic.980

**Published:** 2014-08-28

**Authors:** Noreen Kearns, Liam Coen

**Affiliations:** UNESCO Child & Family Research Centre, School of Political Science & Sociology, National University of Ireland, Galway, Ireland; Research and Library Service, Houses of the Oireachtas, Dublin, Ireland

**Keywords:** domestic violence, Ireland, integration, coordination, joined-up, ecological, health sector, justice sector

## Abstract

**Introduction:**

This paper assesses the policy developments pertaining to the implementation of an integrated approach to domestic violence over the past 15 years. The contextual setting is outlined in terms of the international policy response to the problem of domestic violence based on an ecological perspective.

**Description of policy and case:**

Periods of core strategic policy and related structural developments are considered illustrating the Irish experience of domestic violence policy-making and service provision. The value of adopting an integrated approach to domestic violence based on the rationale of improving strategic policy formulation, coordinating service provision and facilitating joined-up governance is set out. The core facilitators and challenges associated with such an approach are described.

**Analysis and conclusion:**

The policy framework and restructured landscape of domestic violence in Ireland has undergone significant change over the past decade and a half. The paper uses a three-dimensional matrix of domestic violence policy development and service integration as a means of addressing horizontal, vertical and resource aspects of collaboration and integration. While the changes have been characterised by significant phases of fluctuation in terms of coordinated action and the situation currently appears promising, however it is too early to judge the outcomes of the most recent reforms.

## Introduction

Some of the most difficult public policy problems of the modern era have been described as complex, intractable, open-ended and ‘wicked’ [1]. It is increasingly acknowledged that such problems are comprised of underlying societal and environmental determinants, and cannot be adequately addressed in an autonomous, unitary manner. The contention is that the complex array of socio-environmental factors associated with health and well-being and human activity systems [2] requires an integrated response by multiple actors working across and between organisations, institutions, agencies and sectors. Domestic violence could be viewed as a ‘wicked problem’ [3] based on its nature and the issues involved in addressing it. One of the key challenges associated with addressing the problem of domestic violence is developing appropriate public policy and effective intervention strategies [4]. In recognition of this multifaceted problem, one of the most commonly applied approaches to inform domestic violence research is Bronfenbrenner's ecological theory of human development [5]. This highlights interactions between individuals and their multiple, interconnected environments, and comprises factors at individual (personal characteristics and socio-demographic factors), micro-system (informal supports of individuals), meso-system (informal supports between individuals and systems), exo-system (formal organisations and social systems) and macro-system (cultural/normative context) levels. This broad-based conceptualisation has been adapted by Heise [6] to explain the risk factors and underlying causes of violence against women. Heise [6] sought to reconcile the individual-factor perspective with the sociocultural perspective, using an ecological framework to integrate the array of aspects at personal/individual, interpersonal/situational, community/institutional and societal/cultural levels.

In recent years, a number of prominent international institutional actors have raised the profile of integrated, collaborative approaches involving joint initiatives to address the issue. UNICEF [7, p. 45] highlighted the need for a systematic approach involving a multiplicity of sectors including the judicial system, the media, educators, health care authorities and governmental and non-governmental agencies in addressing the problem of violence against women. The World Health Organisation adopts an ecological model for understanding the causes, consequences and prevention of violence. It outlines strategies for working with multiple systems and settings, specifically, suggesting the use of a wide variety of tactics, such as policy change, organisational change, systems advocacy, media campaigns and rape awareness/prevention education, to create broad-based systemic change [6, 8]. Several of the World Health Organisation's recommendations [9] and a Council of Europe report [10] indicate the complexity of issues to be addressed and reflect the need for multi-sectoral and collaborative approaches to tackle domestic violence. Such approaches to domestic violence are now common place across several countries worldwide with examples of such initiatives in the United Kingdom, the United States, Canada, Australia, New Zealand, Africa, Asia and Latin America [11].

At the core of the move towards greater coordination amongst the plethora of bodies involved directly and indirectly in domestic violence is the rationale of improved policy development, prevention strategies and service delivery. Also implicit in this is a shift to governance and an acknowledgement of the role of non-governmental actors in the policy process [12, 13]. Partnership and related terms such as joined-up government, joined-up governance and inter-agency working have been deployed to denote ‘the aspiration to achieve horizontally and vertically coordinated thinking and action’ [14, p. 35]. Ultimately, joined-up approaches involve bringing together public, private and voluntary bodies to work across organisational boundaries towards a common goal and are frequently employed as a means of tackling complex or wicked problems [15].

This paper aims to shed light on developments concerning the adoption of a collaborative, integrative approach in the domestic violence arena. It takes the case of the Republic of Ireland (hereafter Ireland), assessing the trajectory of change in the health and justice sectors which has characterised domestic violence over the past decade and a half. It outlines significant phases of fluctuation involving policy, legislative, strategic and structural developments. The paper applies Browne et al.'s [16] integration model as a means of addressing the challenges associated with collaboration and facilitating a more effective response to the problem. By tracing the significant milestones in the Irish domestic violence arena and conceptually examining policy development and service integration, we hope this paper will add to the knowledge of integrative working across multiple sectors by examining what facilitates and hinders collaboration in the domestic violence field.

## Tracing Ireland's trajectory of domestic violence policy development and change in the health and justice sectors since the mid-1990s

In the following, we consider the major policy initiatives at the macro-level within the health and justice sectors which have primary responsibility for domestic violence in Ireland. The position of the health sector to respond to domestic violence through its primary and secondary health and social services is unique on the basis that the health care system is the most common route through which those experiencing or at risk of domestic violence present themselves. Moreover, as well as its frontline role in service provision, the health sector is also a significant funding and resourcing body for other agencies providing domestic violence supports and services. Up to 2005, the Department of Health and Children was the body responsible for the provision of health and social services to victims of domestic violence through the (former) regional health boards. Some of its key functions included responsibility for the administration of funding and service provision in this area. These functions and structures were subsumed by a new agency, the Health Service Executive, following its establishment in 2005.

A noteworthy milestone in the domestic violence policy trajectory was the establishment by government of a Task Force on Violence Against Women in 1996. Its purpose was to develop a coordinated response and strategy regarding the problem of domestic violence in Ireland [17–19]. Its membership comprised civil servants, experts drawn from practice and academia, and public servants from the health and police services. The publication of the Report of the Task Force on Violence Against Women [17] represented a major policy development signifying government's commitment to addressing the problem. The report identified the significant role of the health and social services sector in coordinating and providing domestic violence services, and highlighted the need to improve service provision.

One of the recommendations of the Task Force Report was to set up a National Steering Committee on Violence against Women to coordinate services and policies at the national level [18]. The subsequent establishment of this committee in December 1997 in the Department of Justice and Equality represented an important development in the domestic violence arena. Its membership comprises representatives from the statutory and voluntary sectors and Regional Committee structures representing health, justice and local government and non-governmental organisation sectors. Its core focus has been to address the lack of a strategic approach to the problem of violence against women. Indicative of such work were the recommendations of an Inter-Departmental Sub-Committee of the National Steering Committee for the coordination of service delivery and funding decisions; the coordination of service needs and plans; increased information on funding levels and availability; and the establishment of clear budget lines for addressing the problem across relevant government departments [18].

Another noteworthy structural outcome of the Task Force Report was the establishment of eight Regional Planning Committees at the end of 1998, one in each of the former health board areas. The remit of the Planning Committees included assessing needs in the region; developing local plans to meet those needs; and developing an implementation plan and service targets. Since 2009, they have been replaced by eight Regional Advisory Committees, with inter-sectoral membership drawn from state organisations and relevant non-governmental organisations. A key remit of the Advisory Committees is to foster inter-sectoral collaboration and partnership working within and between the statutory and non-statutory service providers in the planning and delivery of domestic violence services at regional level and implementing national strategic policy [18–20]. This Regional Committee structure feeds into national policy development through representative membership in the National Steering Committee on Violence against Women.

In 2010, the Health Service Executive published a policy on domestic, sexual and gender-based violence for the health sector [19]. Such a move was in recognition of the absence of national planning across the health services in Ireland up to this point to guide the work of Health Service Executive staff and others commissioned by the Health Service Executive to provide services for victims of domestic violence. The policy aims to prevent domestic and/or sexual violence, and to provide a continuum of supports to those families experiencing or at risk of such violence [19, p. 9]. It also intends to foster an increased understanding of domestic violence, particularly amongst frontline staff through training and awareness raising.

Another key player in the domestic violence arena is the justice sector. The Department of Justice and Equality is responsible for legislative instruments in this area, the development of perpetrator programmes and overseeing the police force. The Domestic Violence Act 1996 was the first specific legislative instrument on domestic violence in Ireland [21]. The Garda Síochána's (Irish police force) policy on domestic violence was first published in 1994 and subsequently revised in 1997 and 2007 [22]. The Garda Síochána is responsible for a Domestic Violence Unit charged with developing and implementing the policy by coordinating and monitoring the police response to domestic violence incidents throughout the country. The Garda Síochána has a representation on both the National Steering Committee on Violence against Women and Regional Advisory Committees [18].

In 2007, the Department of Justice and Equality published The National Women's Strategy 2007–2016 [23]. Several of the inter-sectoral actions contained in the strategy mirror a series of gaps highlighted in the Council of Europe report [10] on governmental responses to the implementation of Recommendation Rec (2002) 5 on the protection of women against violence. One of the most significant contributions of the strategy was a commitment to establishing a national executive office the intended purpose of which would be to ensure the delivery of a well-coordinated, ‘whole-of-government’ response to violence against women and domestic violence [18]. The National Office for the Prevention of Domestic, Sexual and Gender-based Violence, known as Cosc, was subsequently established in June 2007.

Cosc's strategic remit entails enhancing the coordination of policies and services for domestic, sexual and gender-based violence across the country. Since its inception, it has pioneered a series of significant strategic and structural developments in this arena. One of Cosc's most salient outputs to date is the publication of a five-year national strategy (2010–2014) to address domestic, sexual and gender-based violence [18]. The strategy is underpinned by a model of primary and secondary intervention. The former is concerned with preventing the problem from occurring or, when it has taken place, to prevent its recurrence through awareness raising, education and attitudinal change. The latter involves a spectrum of responses ranging from routine enquiry and screening in General Practice or hospital settings with the aim of facilitating disclosure, to direct service provision for victims regarding accommodation, counselling and medical attention, and relief provided through the justice process [18]. At the core of the strategy is the principle of collaborative working, with a strong emphasis on the need for action to strengthen intra- and inter-organisational coordination across statutory and non-governmental organisation sectors and relevant frontline services [18]. Evidence of Cosc's collaborative work is underpinned by its membership in, or chairing of, several committees, organisations and agencies in the domestic violence field. The National Steering Committee on Violence against Women now comes under the remit of Cosc. The Department of Justice and Equality in conjunction with the Law Reform Commission are currently reviewing the aspects of the law on domestic violence.

## Discussion of Irish domestic violence policy reform intentions, achievements and challenges

The institutional environment pertaining to domestic violence in Ireland has evolved considerably over the past 15 years. This is based on a recognition of the need for a multi-agency response including training, campaigning, legal and emotional advocacy and support services to assist victims or potential victims to engage with the health and justice systems and with other voluntary and statutory sector agencies [24]. Over this period, there has been strong evidence of a move towards a more focused, strategic approach to the problem both nationally and regionally, underpinned by an ecological approach comprising the collaboration of multiple sectors and agencies involved in policy planning and service delivery. Some of the most noteworthy highlights during this period include the passing of the first dedicated piece of legislation to address domestic violence in Ireland in 1996, the setting up of the Task Force on Violence Against Women in 1996, and the publication of its report in 1997 [17]. This led to the establishment of the National Steering Committee on Violence against Women in late 1997 and the formation of the Regional Planning Committees (now Regional Advisory Committees) at the end of 1998.

While the developments during this period signified an important advancement in terms of addressing the problem of domestic violence in Ireland, the intervening decade up to 2007 was characterised by a lull in terms of coordinated policy and operational service development. A distinct policy-service gap occurred with regard to the implementation of initiatives and recommendations contained in the 1997 Task Force Report [17]. The failure to develop a national strategy and action plan on violence against women during this period posed a particular challenge for services and professionals working in the area of domestic violence. A report published by the Women's Health Council in 2007 [25] pointed out that despite funding increases over the years, many services continue to struggle in coping with demand. It also noted that while the work of the Planning Committees included some excellent initiatives, there were significant local variations, resulting in an ad hoc system of planning and delivery. The report critiqued the lack of a national systematic approach by the health sector in providing its own services, as well as poor linking with other statutory and non-statutory service providers. Moreover, whilst the problem of domestic violence is mentioned in the national health strategy [26], it is not addressed in a number of other national strategic policy documents in the areas of health promotion, primary care and mental health [25]. A major gap in tackling the problem at the national level, leading to a dearth of strategic and service planning and provision for domestic violence, was similarly recognised in the national strategies of Cosc and the Health Service Executive [18, 19]. In reference to the lack of an integrated approach to domestic violence in Ireland over several decades, Cosc [18] highlighted the need for systematic change at individual, organisational, societal and national policy levels. Such a change would help ensure that those involved in tackling the problem are fully aware of best practice in this field. It also referred to a need for an attitudinal/cultural change regarding abuse and violence, and for a change at the national policy level so that countrywide action would produce the most effective response to the problem.

In contrast to the above rather static period, Ireland is currently at a critical peak stage in terms of implementing an integrated, ecological approach to the problem of domestic violence. Testament to this are a number of important recent structural and policy developments, most notably the establishment of the national executive office Cosc, and recently published policies, strategies and plans by Cosc, the Health Service Executive, the Department of Justice and Equality and the Gardaí, all of which deal with developing a coordinated response to the problem. There are significant benefits of horizontal and vertical coordination including better policy-making and use of scare resources, synergistic working amongst key stakeholders and the provision of a set of seamless services for citizens [14]. In order for joined-up governance to occur, deliberate planning entailing the specification of goals and objectives and clarification of roles and responsibilities across the different stages and forms of coordination work is necessary [27]. Ultimately, what is required is a supporting architecture which resets incentives, provides authority, builds long-term trusting-based relationships and recognises and rewards cooperative behaviours in order to operate a joined-up model [15].

Notwithstanding the complexity of domestic violence and the multiple forms it can take within the family, the services for this problem often operate independently [28]. This is in line with the prevalent model of public policy-making which reduces complex problems into separate, rationally managed components [2]. Conceptualising domestic violence as a wicked problem requires a change in the way of thinking about how the problem is to be tackled [29]. Best practice in the field asserts that a complex response comprising multi-agency working is the most effective means of dealing with this complex issue. In terms of implementing a multi-agency approach [24] in the Irish context, significant challenges arise. If the various government departments, sectors, agencies and organisations with the responsibility for domestic violence policy formulation and implementation are considered, a potential ‘administrative jungle’ emerges, with different bodies working along different national, regional and local territories. The health system was divided into four regions during the reforms of the mid-2000s and is currently undergoing a further restructuring process. The police service operates on a different regional basis than that of the health service, and has both local and regional offices. A myriad of specialist domestic violence organisations in the non-governmental organisation sector operate individually as part of either formal national networks such as Women's Aid, the Rape Crisis Network Ireland and SAFE Ireland, or loosely coupled networks such as Counselling Services, Women's Refuges and Support Services. Such structural challenges were highlighted by Cosc [18] in terms of achieving horizontal coordination amongst organisations located in different systems such as justice and health. It also pointed to difficulties associated with vertical coordination between local, regional and national levels, and low levels of coordination between the State and non-governmental organisations [18].

## Coordination based on a three-dimensional matrix of domestic violence policy development and service integration

Hence, whilst the current indications of collaborative working in the Irish domestic violence arena are positive, there are several potential challenges to be addressed with respect to strategic policy development and service integration. A core question is how can a joined-up partnership approach to domestic violence be facilitated and what lessons can be learned from the relevant literature? Browne et al. [16] developed a model of human services integration based on the value of adopting a coordinated approach amongst sectors, agencies and disciplines in terms of achieving a unified and effective mix of services. The model comprises sectoral integration on the vertical axis, universal, targeted and clinical types of services on the horizontal axis, and core public, private, non-profit or voluntary funding and other resources on the third axis. A three-dimensional matrix based on Browne et al.'s [16] integration model could be usefully applied as a means of addressing various collaborative challenges and facilitating policy, service and resource integration in the domestic violence arena. The model requires a need to clarify the roles and relationships across national, regional and local levels such as the justice, health, education and social services sectors, and upwards and downwards between government departments in particular, health and justice, and non-governmental organisations. It also requires integrated service provision along a continuum of primary and secondary levels, and formal structures for coordinating the main sources of funding and other resources across relevant departments and agencies if true joined-up governance is to be achieved.

At the national level, willingness and drive from the political centre to be present to ensure that vertical as well as horizontal coordination occurs is of central importance [30]. This involves not simply an infrastructure of coordination but entails an architecture composed of a set of processes and structures and a cultural appreciation of values and power, alongside an awareness of bureaucratic politics and organisational practices to facilitate implementation. Moreover, the requirement for real cooperation is a major challenge and is dependent on the behaviour of politicians to take action, and involves central government negotiating and persuading a wide range of organisations in public and non-governmental sectors to embrace joined-up governance [14].

At regional and local levels, commonly cited structural and interpersonal barriers encountered in the domestic violence field are similar to those challenges found in the literature on joined-up or partnership working. These include differing perceptions of goals, roles and responsibilities; leadership; lack of involvement and marginalisation of voluntary sector services; power and relationship differentials; professional value conflict and cultural clashes; communication problems; resourcing issues; lack of commitment and trust; resistance to change; and lack of time [11, 31–35]. Still, some barriers appear more specifically in the service integration literature including large caseloads; limited service offerings; lack of complete information; unawareness of overlapping services; gaps in screening and diagnostic services; little consideration of political bases; and lack of resources [36–39]. Formal structures facilitating integration include networks, committees and coalitions of local agencies, organisations and possible funders charged with the responsibility for organising and delivering local human services [16]. An awareness of and ability to address power differences between agencies is also important [40].

Finally, differences in bureaucratic structures, levels of expertise, funding mechanisms and regulations can complicate organisational integration [16]. Implementing joined-up policies in a joined-up manner implies service integration, or at the least, service coordination [32]. Frontline domestic and sexual violence support services play an important role in coordinating their clients’ linking in with, or access to other services [41, p. 71]. The responses of professionals are crucial, and they have much to contribute to the policy process by way of relaying service users’ experiences as well as their own professional knowledge. Outreach and informal contacts and networks formed through communities of frontline service providers working with community groups, agencies and programmes in this area are important mechanisms enabling cross- and intra-sectoral collaboration, and a continuum of service provision [16, 24, 42]. Multi-agency referral systems and staff training and support are also important [24].

## Conclusion

The ecological framework aids understanding of the complexity and dynamics of domestic violence by taking account of the interrelated settings and levels in which individuals exist (individual, interpersonal and socio-cultural) and the various risk and protective factors across these [28, 43, 44]. It has integration at its core and requires action at vertical and horizontal levels, across sectoral and agency boundaries, primary and secondary levels of service activity and resource pooling. This paper has sought to illustrate some of the most significant policy developments particularly in the health and justice sectors in addressing the problem of domestic violence in Ireland over the past 15 years. It has highlighted the complexity of issues which pose a significant challenge for the achievement of an integrated, joined-up response to domestic violence policy formulation and service provision. Such challenges are associated with micro-, meso-, exo- and macro-system factors comprising structural and cultural differences amongst statutory and non-statutory sectors and the varying professional, ideological and practice backgrounds, experiences and attitudes of those working directly or indirectly in the area of domestic violence.

In applying Browne et al.'s [16] model, this paper has considered the implications for addressing the wicked problem of domestic violence, requiring integration to occur vertically across the multiple sectors involved both directly and indirectly in domestic violence including health, justice and education. Moreover, a close working relationship by the statutory sector with domestic violence organisations and support services in the non-governmental organisation sector is necessary if integration is to produce the right outcomes for victims and their families. Coordination also needs to occur horizontally, at the primary and secondary stages of service provision (prevention, early intervention and tertiary) in order to provide a continuum of services and programmes across various disciplines. Finally, effective partnership working requires the pooling of resources across sectors, agencies and disciplines. The integrated policy framework and restructured landscape of domestic violence service provision in Ireland remains at an early stage of development, and whilst current indications are relatively positive, future research will be necessary in order to assess the implementation and outcomes of the joined-up moves over the coming years.
